# Quantum Chemical Model Calculations of Adhesion and Dissociation between Epoxy Resin and Si-Containing Molecules

**DOI:** 10.3390/molecules29215050

**Published:** 2024-10-25

**Authors:** Hao Xue, Yingxiao Xi, Naoki Kishimoto

**Affiliations:** Department of Chemistry, Graduate School of Science, Tohoku University, 6-3, Aoba, Aramaki, Aoba-ku, Sendai 980-8578, Japan; xue.hao.p2@dc.tohoku.ac.jp (H.X.); xi.yingxiao.r3@dc.tohoku.ac.jp (Y.X.)

**Keywords:** epoxy resin, silicon hydroxide, quantum chemical calculation, adhesion, dissociation

## Abstract

There is no doubt that when solid surfaces are modified, the functional groups and atoms directly bonded to solid atoms play a major role in adsorption interactions with molecules or resins. In this study, the adhesion and dissociation between epoxy resin and molecules containing Si atoms were analyzed. The analysis, conducted in contact with the solid surface of silicon, utilized quantum chemical calculations based on a molecular model. We compared some Si-containing molecular models to test quantum chemical calculations that contribute to the study of adhesion and dissociation between epoxy resins and solid surfaces somehow other than simple potential energy curve calculations. The AFIR (artificial force induced reaction) method, implemented in the GRRM (global reaction route mapping) program, was employed to separate an epoxy resin model molecule and three types of silicon compounds (Si(CH_3_)_2_(OH)_2_, Si(CH_3_)_4_, and (CH_3_)_2_SiF_2_) in three directions, determining their minimum dissociation energy when changing the applied energy by 2.5 kJ/mol. In systems with weak hydrogen bonds, such as Si(CH_3_)_4_ or (CH_3_)_2_SiF_2_, the energy required for dissociation was not large; however, in systems with strong hydrogen bonds, such as Si(CH_3_)_2_(OH)_2_, dissociation was more difficult in the vertical direction. Although anisotropy due to hydroxyl groups was calculated in the horizontal direction, dissociation remained relatively easy.

## 1. Introduction

This study explored the durability improvement of epoxy resins when used as sealing materials for oxidized metal components. Epoxy resins, owing to their excellent mechanical properties, have become indispensable in various high-performance engineering fields, including aerospace, marine, and automotive industries [1,2,3]. Properties such as high modulus, strength, low creep, and thermal stability are largely attributed to the highly cross-linked microstructure of epoxy resins, which provides the rigidity necessary for structural applications [4,5,6]. Our previous research [7] examined the formation of cross-linked network structures and the physical properties of epoxy resins. However, while the cross-linked structure imparts strength and stiffness, it also introduces brittleness, significantly limiting the material’s resistance to crack propagation [8]. This brittleness restricts the broader use of epoxy resins in load-bearing applications, confining their roles predominantly to adhesives and matrix materials in fiber composites.

To address the brittleness of epoxy resins, researchers have focused on incorporating various fillers to enhance their fracture toughness. Among these fillers, silicon dioxide (SiO_2_), particularly in its oxide form, has garnered significant attention due to its unique properties and versatility, which contribute to the overall performance of composites [9,10,11,12]. Additionally, its interaction with environmental moisture enhances its effectiveness, as silicon-based materials like SiO_2_ are commonly used for surface passivation and adhesion enhancement. When exposed to moisture, SiO_2_ surfaces generate hydroxyl groups, which promote hydrogen bonding and improve interfacial adhesion with polymer matrices [13,14]. These hydroxyl groups significantly alter the surface properties, forming hydrogen bond networks that enable a wide range of applications [15]. In this study, we investigated adhesion and dissociation processes at interfaces involving hydroxyl-rich silicon surfaces. To represent these surfaces, we used Si(CH_3_)_2_(OH)_2_ as a simplified model. Although the presence of methyl groups in Si(CH_3_)_2_(OH)_2_ does not match the exact composition of oxidized SiO_2_, this molecule preserved the essential characteristics of hydroxylated surfaces. This allowed us to study interfacial hydrogen bonding and adhesion behavior efficiently within a controlled computational framework. Although molecular dynamics (MDs) simulations have significantly advanced our understanding, they often fail to capture the full extent of quantum-mechanical phenomena. For instance, Wang et al. [16] and Li et al. [17] used MDs simulations to investigate the structural, volumetric, and dynamic properties of interphase regions, as well as the effects of interfacial water and SiO_2_ surface wettability on adhesion in epoxy nanocomposites. Although these studies provided valuable insights, the molecular-level intricacies of the adhesion and dissociation processes between epoxy resin and silicon hydroxide require further exploration. Quantum chemical calculations have been proposed to address this gap, offering precise insights into the molecular forces governing these interactions. The utility of quantum chemical calculations in understanding complex molecular interactions is well established. For instance, Oya et al. [18] employed first-principles calculations using the global reaction route mapping (GRRM) program [19] (ver. 20, HPC Systems Inc.) to analyze the crosslinking process of epoxy and to investigate how branching structures influence the thermal and mechanical properties.

This study employed the artificial force induced reaction (AFIR) method [20,21] within the GRRM program to systematically investigate molecular interactions at the epoxy–SiOH interface, with a focus on adhesion and dissociation processes. The AFIR method, a sophisticated tool in quantum chemistry, facilitates the exploration of reaction pathways by applying artificial forces that lower reaction barriers. This approach enables the identification of bonding and dissociation events that may be elusive under natural conditions, making it particularly effective for dissecting the complex molecular dynamics governing epoxy–SiOH interactions. The analysis emphasized the role of hydroxyl groups on the SiOH surface, as these groups significantly influence hydrogen bonding, electrostatic interactions, and overall adhesion strength. Additionally, computational analyses were conducted at the B3LYP/6-31G level of density functional theory (DFT) to simulate interactions between Si(CH_3_)_2_(OH)_2_ and tertiary amine products derived from epoxy and amine twice reactions. We optimized structures that represent varying degrees of hydrogen bonding to calculate the dissociation energies required for Si(CH_3_)_2_(OH)_2_ to detach from these complexes, using the AFIR method to quantify the forces involved in these separation processes. Furthermore, computational analysis was extended to calculate dissociation energies for Si(CH_3_)_4_ and (CH_3_)_2_SiF_2_ when interacting with the product molecules, enhancing our understanding of how different silicon compounds influence the adhesive properties of the interfaces. This study’s results will guide the development of more durable sealing materials, improving the performance of epoxy resins in applications that require bonding with oxidized metal surfaces. The results are expected to provide valuable insights for designing epoxy resins to ensure their long-term performance and strong adhesion in challenging environments.

## 2. Results

**Figure 1:**
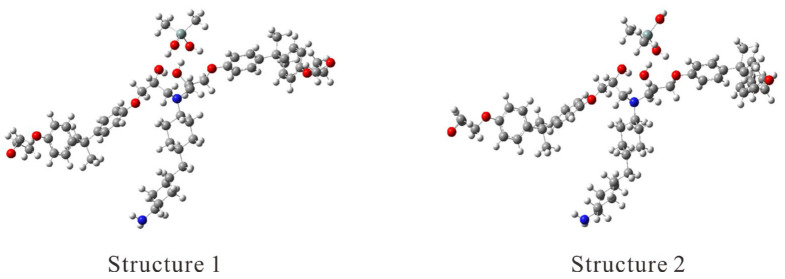
Two hydrogen-bonded structures of Si(OH)_2_(CH_3_)_2_ and a tertiary amine product molecule (DGEBA-PACM-DGEBA). Atoms are distinguished by color (H: white, C: gray, N: blue, O: red, Si: greenish gray).

The enthalpy of Structure 1 was 280.14 meV (6.46 kcal/mol = 27.03 kJ/mol), which was more stable than that of Structure 2 at 0 K. This energy accounted for the correction for zero-point vibrational energy. Structures 1 and 2 are illustrated in Figure 1.

**Figure 2:**
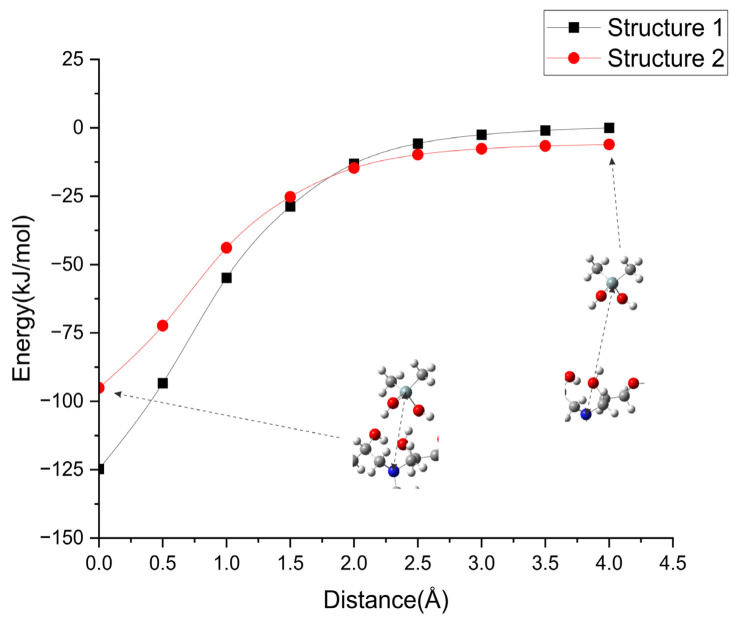
Potential energy curves as a function of the distance between Si and N (nearer) for Structures 1 and 2. The distance 0.0 corresponds to the equilibrium length.

In AFIR calculations, a stable equilibrium structure is achieved when the added negative energy is small, starting from an equilibrium structure. However, if the added negative energy exceeds the activation energy barrier of the dissociation reaction pathway, an equilibrium structure cannot be reached. In such a case, the inter-fragment distance increases, leading the GRRM program to determine dissociation. We investigated the dissociation of Structures 1 and 2 by adding energy to separate them. For comparison, we calculated the potential energy curves when the rigid molecular fragments moved apart (Figure 2). The distance of 0 in Figure 2 corresponds to 5.62 Å and 5.70 Å for the length of Si-N(nearer) in Structures 1 and 2, respectively. The energy 0 in Figure 2 corresponds to the interaction energy of Structure 1 at the distance 4.0 Å (equivalent to 9.62 Å between Si and N (nearer)), while the relative energy for Structure 2 at this distance was −6.1 kJ/mol.

**Figure 3:**
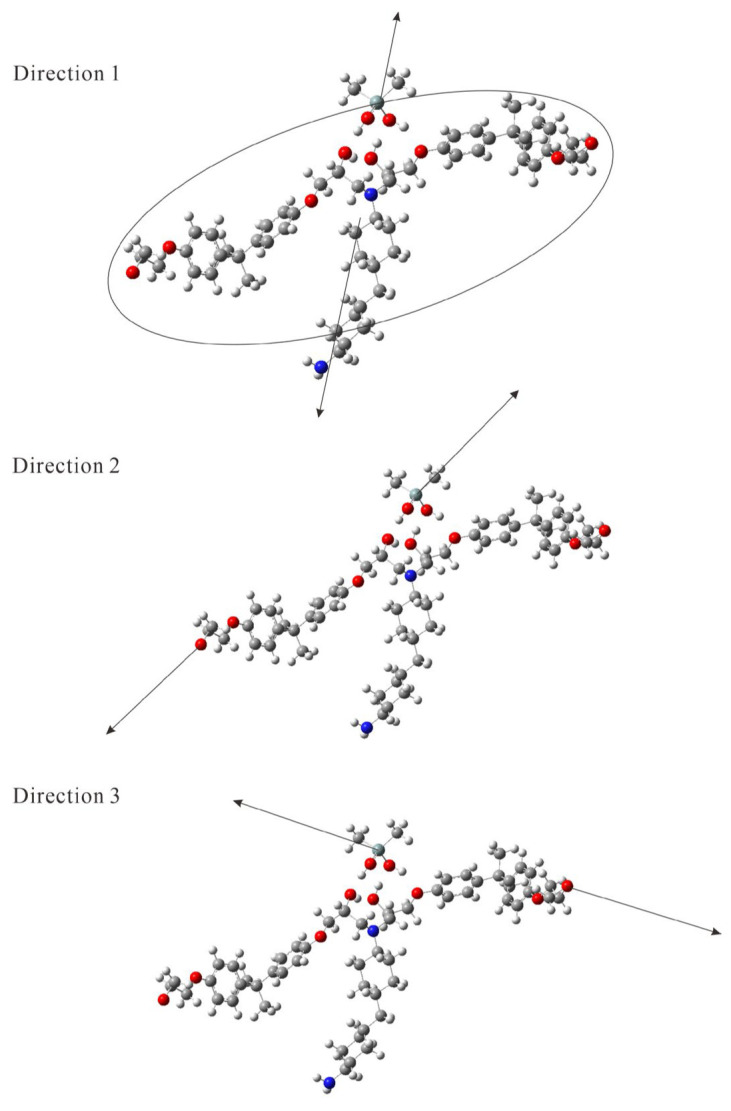
Three directions in the AFIR calculation for Structure 1. Direction 1 shows the repulsive direction between the Si atom and all atoms in the tertiary amine product molecule. Directions 2 and 3 show repulsive directions between the Si atom and an oxygen atom in the two epoxy groups.

We varied the energy of dissociation in the AFIR method in increments of 2.5 kJ/mol to investigate the energy at which the GRRM program determined that dissociation occurred beyond the activation energy. We aimed to vary the direction of dissociation by specifying the fragment group or atom, with the three calculated dissociation directions shown in Figure 3. In Direction 1, all atoms in the Si-containing molecules were fixed as fragment A, while all 139 atoms in the tertiary amine product molecule (DGEBA-PACM-DGEBA) were included in fragment B. As the DGEBA-PACM-DGEBA molecule has a two-dimensional structure, the direction in which the atoms in this molecule were pulled was limited. In Directions 2 and 3, the central Si atom was fixed as fragment A, and one of the unreacted oxygen atoms in two terminal epoxy groups in DGEBA of DGEBA-PACM-DGEBA was set as fragment B. The calculated threshold energy (minimum energy) values for dissociation along Directions 1–3 are listed in Table 1. Fragments A and B dissociated at energies greater than or equal to those indicated in Table 1. To compare the minimum energy for dissociation involving hydrogen bonds of hydroxy groups with other cases, we conducted AFIR calculations for Si(CH_3_)_4_ and (CH_3_)_2_SiF_2_ with DGEBA-PACM-DGEBA, as listed in Table 1. All structural coordinates of the equilibrium structures are provided in the Supporting Information. Notably, obtaining the transition structure on the reaction path leading to the dissociation channel from the equilibrium structure of a hydrogen-bonded system involving multiple hydroxy groups was difficult. As a result, we could not estimate the activation energy of dissociation in this study of the giant molecular system using the conventional reaction path search method with the GRRM program.

**Figure 4:**
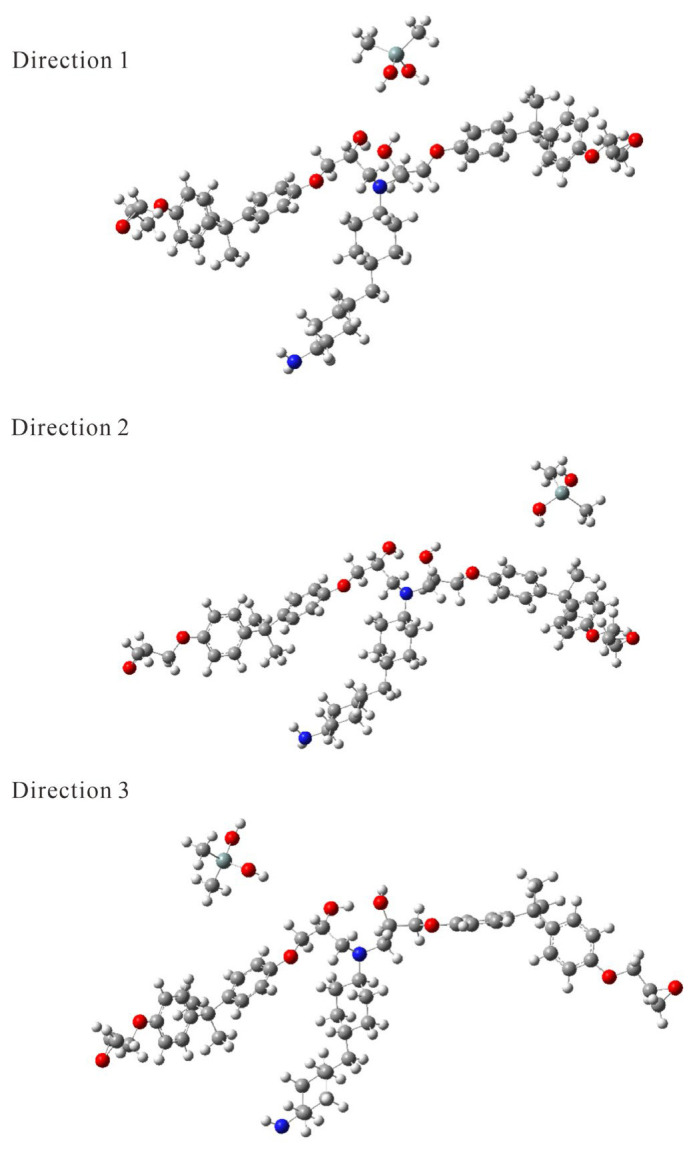
Dissociated structures at Directions 1–3 in the AFIR calculation for Structure 1.

The dissociated structures at the three directions are presented in Figure 4. The Si-N (nearer) distance was measured at 7.48 Å when 35 kJ/mol was added in Direction 1 to Structure 1 during AFIR calculations. In the GRRM program, dissociation was determined when the nearest neighbor distance criteria, proportional to the sum of the atomic radii (*r*_H_ = 1.138 Å, *r*_O_ = 1.236 Å, and *r*_F_ = 1.433 Å), were exceeded. Table 1 lists the values calculated using the dissociation criterion for the standard valence bond case (default values: *UpDC* = 10, *DownDC* = 8) [22].

Although we adjusted the dissociation criterion in the GRRM program for hydrogen bonds (*UpDC* = 12, *DownDC* = 12) to be larger than for valence bonds, results for Structures 1 and 2 were nearly identical to those in Table 1. Table 2 presents results for the hydrogen bonding option, noting only two increases in the energy value (Direction 3 for Structure 2 and (CH_3_)_2_SiF_2_). Regarding Structure 1 and Si(CH_3_)_4_, we confirmed that results were consistent with those in Table 1.

In order to investigate the effect of the basis function in AFIR quantum chemical calculations for dissociation judgment, we used 6-31G(d) basis set with polarization function. Table 3 represents the results for Si(OH)_2_(CH_3_)_2_. The number of basis functions in the 6-31G(d) case was about 1.53 (=1218/798) times larger than in the 6-31G case. When we estimated the calculation time as the third to fourth power of the number of basis functions, the DFT calculation time with 6-31G(d) was 3.55 to 5.42 times (=1.53^3^ to 1.53^4^ times) longer than that for 6-31G. Obtained results with the 6-31G(d) basis set are mentioned in the Discussion.

## 3. Calculation Methods

In the quantum chemical calculations, the DFT hybrid functional B3LYP, which is the most widely employed functional, and the 6-31G basis set were employed. Initially, several hydrogen-bonded systems were created between Si(CH_3_)_2_(OH)_2_ and a tertiary amine product derived from two identical epoxy molecules—DGEBA (bisphenol A diglycidyl ether) and one amine molecule (PACM, 4,4′-methylenebis(cyclohexylamine))—using the AFIR method. Two different structures were chosen from the stable structures obtained from interactions from random directions: (1) a fully hydrogen-bonded system between two hydroxyl groups in Si(CH_3_)_2_(OH)_2_ and two hydroxy groups in the reaction product, and (2) a half-hydrogen-bonded system between one hydroxy group in Si(CH_3_)_2_(OH)_2_ and one hydroxyl group in the reaction product. The equilibrium structures (EQs) of the two supermolecules were determined through structural optimization calculations (Supplementary Materials).

To estimate the energy for the dissociation of Si(CH_3_)_2_(OH)_2_ from EQs 1 and 2, quantum chemical calculations (B3LYP/6-31G) were used to test dissociation in various directions by adjusting the energy at which the molecule was pulled apart, using the AFIR method. The structural relaxation was calculated by adding minus energy to the two molecules and performing structural optimization, and if the distance between them exceeded a criterion, the two were determined to have dissociated. The atoms in molecular fragments where force was applied were specified, and the dissociation energy was estimated by gradually increasing the energy until the GRRM program indicated that the two fragments had dissociated. Additionally, for comparison with hydrogen bonds involving hydroxy groups, the dissociation energies of Si(CH_3_)_4_ and (CH_3_)_2_SiF_2_ with the product molecules were calculated using the AFIR method.

## 4. Discussion

In the case of the reference material Si(CH_3_)_4_, the interaction calculated in Table 1 was extremely weak (<2.5 kJ/mol), resulting in poor anisotropy. The small difference in electronegativity (χ) between C (χ^C^ = 2.55) and H (χ^H^ = 2.20), according to the textbook Pauling values, led to only a weak interaction between -CH_3_ in Si(CH_3_)_4_ and the hydroxyl groups in the epoxy resin. Consequently, it can be concluded that methylated epoxy resin on the Si substrate surface was highly ineffective in adsorbing the resin and easily peeled off.

**Figure 5:**
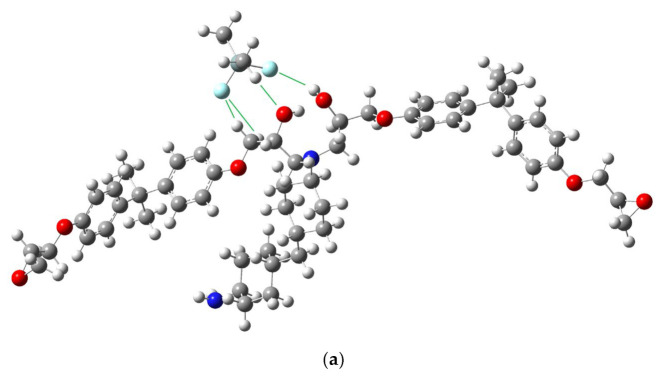

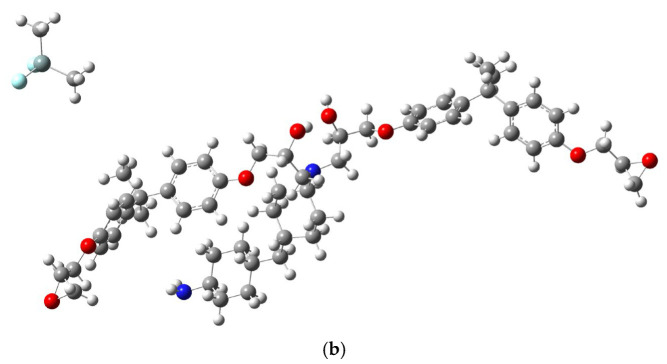
(**a**) The calculated equilibrium structure in the case of (CH_3_)_2_SiF_2_ at Direction 3 in the AFIR calculation with the added energy of 12.5 kJ/mol and (**b**) the dissociated structure with the added energy of 15 kJ/mol. The dissociation was determined with the criterion for hydrogen bonds. Atoms are distinguished by color (F: light blue).

In the case of (CH_3_)_2_SiF_2_, which contains two fluorine atoms, strong valence bonding was anticipated between Si and F due to the high electronegativity of the F atom (χ^F^ = 3.98) compared to the Si atom (χ^Si^ = 1.90). The strength of these bonds with substrate atoms made peeling off the fluorinated surface more challenging. Additionally, fluorinated metal surfaces are known for their strong non-adhesive properties and low friction, likely due to reduced electron polarization. The calculated results shown in Table 1 indicate that values fell within the range of 7.5 ± 2.5 kJ/mol across the three directions, suggesting that the interaction with the epoxy resin was weak and not highly anisotropic. The substantial difference in electronegativity between Si and F did not facilitate a strong interaction with the hydroxy groups of the epoxy resin, and this study demonstrated that they separate under minimal force. Anisotropy in a horizontal direction increased from 10 kJ/mol to 15 kJ/mol in Direction 3 with the criterion of dissociation, as shown in Table 2. The obtained equilibrium structure at 12.5 kJ/mol and the dissociated structure at 15 kJ/mol are shown in Figure 5, and interaction between the -OH group or the -CH_2_ group and F atoms played a role in maintaining the equilibrium structure at 12.5 kJ/mol. In addition, one -OH group interacted with the methyl group in (CH_3_)_2_SiF_2_. The averaged energy in the horizontal direction was not different from that in the vertical direction. Therefore, when comparing the horizontal and vertical directions for different criterion distances in determining dissociation, it was found that the ease of dissociation in the horizontal direction was mitigated when a molecular model was used. It is interesting to compare these results with the results of the surface model simulations.

In Structures 1 and 2 of Si(CH_3_)_2_(OH)_2_ and DGEBA-PACM-DGEBA, the hydroxy groups exhibited strong interactions known as hydrogen bonds. Each hydrogen bond consists of a hydrogen atom and an oxygen atom, which have a significant difference in electronegativity (χ^O^ = 3.44) compared to H. The calculated potential energy curves indicated interaction energies of ~100 kJ/mol for Structure 2 and 125 kJ/mol for Structure 1. In the AFIR calculations, which evaluated dissociation in a similar direction (Direction 1), the energy required for the dissociation of Structure 1, which had more hydrogen bonds, was higher than that of Structure 2, which had fewer hydrogen bonds.

For horizontal dissociation (Directions 2 and 3) for Si(CH_3_)_2_(OH)_2_, the required energy was lower than for vertical dissociation. As is clear from the tables, there was also a difference in the two horizontal directions, which is thought to be a manifestation of the anisotropy of hydrogen bonding. This AFIR calculation differed from a simple potential curve calculation, as it accounted for energy changes due to the deformation of the DGEBA-PACM-DGEBA segment, as shown in Figure 4. If the hydroxyl groups could rotate freely, the horizontal dissociation energy would likely not differ significantly; however, their rotation was constrained by various interactions within the resin. This constraint is thought to contribute to the observed anisotropy in the calculations. When many hydroxyl groups were present on the resin surface in various orientations and in contact with the solid surface, the horizontal dissociation energy was similar in all directions. Increasing the dissociation criterion and the distance at which dissociation was determined slightly enhanced the anisotropy of the negative energy added to the dissociation of Structure 2, as indicated in Table 2. Consequently, when comparing horizontal and vertical directions, it was found that dissociation was more likely to occur in the horizontal direction, a tendency that remained consistent even with slight changes in the dissociation determination distance. When the polarization function was added to the basis function (6-31G(d)), the pulling energy values were 10 kJ/mol or more smaller than in the case of 6-31G, as shown in Table 3. This means that even though the force pulling the two molecules was smaller than in the 6-31G case, the intermolecular distance was extended to the dissociation limit. This phenomenon was due to the fact that the effective interaction potential energy function of the increasing distance between the two molecules was anharmonically pulled down by the polarization function added to the basis function. It was found that adding a polarization function to the basis function resulted in smaller added energy values than in the case without a polarization function, but the qualitative trend of anisotropy did not change significantly. It should also be noted that results in Table 3 may have been affected by the shift in equilibrium position, as calculations using the 6-31G(d) basis function were started from an equilibrium structure using the 6-31G basis function. We confirmed that the structural change depending on the basis function was small, within 0.1 Å for the Si-N (nearer) distance.

In future investigations, we will employ classical molecular dynamics calculations to study the adhesion and dissociation of epoxy resin and hydroxyl groups on the metal substrate surface. The ability to compare these results with experimental values will largely depend on the accuracy of the constant force used to model hydrogen bonds. Nonetheless, it is crucial to effectively utilize quantum chemical calculations, as demonstrated in this study, to validate the results of classical molecular dynamics calculations on a large scale.

## 5. Conclusions

This study performed quantum chemical calculations for dissociation and separation using an adsorbed molecular model. Several challenges are associated with calculating the simple potential energy curve for dissociating molecular systems, including difficulties in incorporating molecular deformation effects and the inherent subjectivity in determining dissociation based on distance. To address these issues, we performed quantum chemical calculations, including deformation effects, by changing the pulling energy, with dissociation determined according to criteria set by the GRRM calculation program. Notably, when testing the dissociation criteria for hydrogen bonds, the results were comparable to those obtained using default dissociation criteria. The hydroxyl groups in epoxy resins exhibited strong bonding with the hydroxyl groups on solid surfaces, while molecular models indicated that surfaces coated with fluorine atoms or methyl groups detach more easily.

The molecular model revealed that pulling the epoxy resin in the sliding direction on the surface facilitated peeling compared to pulling in the horizontal direction. In forthcoming research, we plan to conduct simulations of the separation between largescale epoxy resin and solid surfaces using classical molecular dynamics methods, discussing the similarities and differences with results obtained from quantum chemical calculations.

## Figures and Tables

**Table 1:** Minimum energy added for dissociation by AFIR calculation along three directions (kJ/mol).

		Direction 1	Direction 2	Direction 3
Si(CH_3_)_2_(OH)_2_	Structure 1	35 *	30 *	25 *
	Structure 2	25 *	12.5 *	17.5 *
Si(CH_3_)_4_		<2.5 **	<2.5 **	<2.5 **
(CH_3_)_2_SiF_2_		7.5 *	5 *	10 *

* Values obtained by calculating in increments of 2.5 kJ/mol. ** The minimum value for dissociation is less than 2.5 kJ/mol.

**Table 2:** Minimum energy added for dissociation by AFIR calculation along three directions with the option for hydrogen bonding (kJ/mol).

		Direction 1	Direction 2	Direction 3
Si(CH_3_)_2_(OH)_2_	Structure 1	35 *	30 *	25 *
	Structure 2	25 *	12.5 *	20 *
Si(CH_3_)_4_		<2.5 **	<2.5 **	<2.5 **
(CH_3_)_2_SiF_2_		7.5 *	5 *	15 *

* Values obtained by calculating in increments of 2.5 kJ/mol. ** The minimum value for dissociation is less than 2.5 kJ/mol.

**Table 3:** Minimum energy added for dissociation by AFIR calculation using B3LYP/6-31G(d) along three directions (kJ/mol).

		Direction 1	Direction 2	Direction 3
Si(CH_3_)_2_(OH)_2_	Structure 1	22.5 *	20 *	7.5 *
	Structure 2	12.5 *	5 *	7.5 *

* Values obtained by calculating in increments of 2.5 kJ/mol.

## Data Availability

Readers can contact the corresponding author.

## Supplementary Materials

The following supporting information can be downloaded at: https://www.mdpi.com/article/10.3390/molecules29215050/s1. Atomic coordinates of interacting equilibrium structures of an epoxy molecule bonded to Si(CH_3_)_2_(OH)_2_, Si(CH_3_)_4_, or (CH_3_)_2_SiF_2_.
