# Supplementary material for: Quantum Chemical Model Calculations of Adhesion and Dissociation between Epoxy Resin and Si-Containing Molecules
Source: Molecules. 2024 Oct 25;29(21):5050. doi: 10.3390/molecules29215050 (PMC11547936; doi:10.3390/molecules29215050)
Supplement: Supplementary file 1 [file molecules-29-05050-s001.zip › molecules-3259059-supplementary.pdf]

Quantum chemical model calculations of adhesion and dissociation between epoxy resin and Si-containing molecules

Hao Xue, Yingxiao Xi and Naoki Kishimoto*

Department of Chemistry, Graduate School of Science, Tohoku University, 6-3, Aoba, Aramaki, Aoba-ku, Sendai 980-8578

*Correspondence: kishimoto@tohoku.ac.jp

Supporting Information

Atomic coordinates of interacting equilibrium structures of an epoxy molecule bonded to $Si(CH_3)_2(OH)_2$, $Si(CH_3)_4$, or $(CH_3)_2SiF_2$.

Structure 1

Si	-5.019723729498	1.982629982654	-3.090456113427
O	-3.560084071125	1.579555242591	-3.975869484315
H	-2.775707457844	2.171328549485	-4.019853225327
O	-4.704888130523	1.792194194203	-1.425070697322
H	-4.340612455150	0.931330832376	-1.069534073728
C	-6.296689491100	0.765651554888	-3.777591867476
H	-7.261777484836	0.890793092002	-3.276025527967
H	-6.434047447006	0.926933570594	-4.851816995965
H	-5.964280164805	-0.266331550446	-3.626663181158
C	-5.457074334476	3.801973323315	-3.370643901797
H	-5.628471726321	4.007777005395	-4.432305916675
H	-6.364718829982	4.058661069572	-2.814620422435
H	-4.652680144584	4.452119585496	-3.011764669513
C	-1.590316031256	-0.135223733279	0.635073763594
H	-1.169627749093	-0.513661378343	1.570878302642
H	-1.866841398867	0.917125750935	0.804782096338
C	-2.886730735811	-0.922446182375	0.365449518526
H	-3.607439364667	-0.676642072892	1.151741279852
C	-2.706675502773	-2.437450200900	0.331479860676
H	-1.951972934313	-2.715175338736	-0.413675248478
H	-3.662604671889	-2.909898170173	0.079836126432
O	-2.277311772685	-2.846480659996	1.665533299655

C	-2.126706128066	-4.203112839410	1.945510710774
C	-2.303510707620	-5.226495201592	1.010668610099
C	-1.772604501519	-4.520489139469	3.266446762109
C	-2.123377591514	-6.562393360595	1.403140781982
H	-2.578540277502	-5.006534584464	-0.013624530192
C	-1.600417714739	-5.849594590184	3.637774101470
H	-1.649174398266	-3.713812497256	3.980058879123
C	-1.770217422496	-6.904805252313	2.714935893140
H	-2.268202213306	-7.333898615552	0.656249425302
H	-1.345227747614	-6.079635098442	4.667475989399
C	-1.511987453759	-8.359723486666	3.170310148635
C	0.018212707353	-8.523410316578	3.385442661830
H	0.387728619978	-7.865412171880	4.178796286642
H	0.277110254231	-9.557542459443	3.640866917078
H	0.544489625866	-8.263487491018	2.461219479080
C	-1.944590367814	-9.395265918346	2.095468306202
H	-1.780157257528	-10.407693146254	2.478590212980
H	-3.006183056645	-9.299498522627	1.845416535977
H	-1.358497783122	-9.283648260111	1.175979140802
C	-2.315741999726	-8.656843503588	4.457304777615
C	-1.750496208236	-9.244222145082	5.596291974551
C	-3.699729628608	-8.377397669695	4.489221844854
C	-2.520813498268	-9.548623425605	6.730331203324
H	-0.692508011877	-9.476172851675	5.625050111458
C	-4.478884599284	-8.675195326231	5.601699978299
H	-4.166638910463	-7.905789692052	3.630505881443
C	-3.888329908422	-9.265223202027	6.729516911133
H	-2.039760962170	-9.997363867554	7.591130348233
H	-5.540420117307	-8.457394118635	5.624033844532
O	-4.748733616797	-9.520874001498	7.798653435102
C	-4.218378768988	-10.169314157478	8.988530406226
H	-3.450991979692	-9.546098709479	9.464861554912
H	-3.769445822857	-11.136144170472	8.725634248665
C	-5.383536951018	-10.359233296129	9.918703227171
H	-6.187394402726	-10.982913934482	9.537800440016
C	-5.693517012848	-9.362717685829	10.963564849283

H	-6.707847646080	-9.273797325722	11.338656134678
H	-5.078243407553	-8.472312104062	11.052214430158
O	-3.513849794616	-0.521849390634	-0.882657147119
O	-5.000315399908	-10.639371468651	11.330665576707
C	-0.293190520387	0.990624029054	-4.053474841222
H	-0.529453347210	0.536575883120	-5.022573382983
H	0.790850697312	1.135620022843	-3.975434746087
O	-0.939128185678	2.308665911654	-3.958430739426
C	-0.389232009045	3.389033466525	-4.670974508017
C	0.531222324727	3.248183781365	-5.709854772965
C	-0.834736989273	4.662440324632	-4.291894818628
C	1.004467529240	4.393244795575	-6.370013946263
H	0.878552123236	2.271719066682	-6.024724313333
C	-0.356054728172	5.785717229078	-4.959452247832
H	-1.537051894786	4.753853415509	-3.470716316824
C	0.574802353659	5.678819652273	-6.014910196505
H	1.717065168627	4.257142477921	-7.174343430587
H	-0.693840655395	6.767874394097	-4.645415283839
C	1.029233980381	6.957322873626	-6.756486425072
C	-0.173080317843	7.462048697780	-7.601638740060
H	-1.025906657760	7.734950994011	-6.971712360589
H	0.105204166993	8.331752058954	-8.207562953949
H	-0.499030277692	6.668778320968	-8.282109906743
C	2.203796708282	6.680577074201	-7.735157087868
H	2.515185230645	7.620076828292	-8.202749741722
H	3.072477709911	6.259973418999	-7.218224107240
H	1.904591967740	5.991306071886	-8.533158025091
C	1.506910660603	8.012565001297	-5.732518206337
C	1.055223928507	9.338398988617	-5.730326744743
C	2.481408542787	7.656140758277	-4.774549973315
C	1.546687174441	10.284487570202	-4.816405822356
H	0.306284184822	9.663145444720	-6.442633062389
C	2.981855053932	8.580217802480	-3.864109175989
H	2.842180688063	6.633056825034	-4.737121121182
C	2.514045847327	9.903228744214	-3.883823568929
H	1.164377588977	11.297643871628	-4.847825936818

H	3.728453012997	8.303608894387	-3.128738666244
O	3.069986294209	10.754805246050	-2.929143991935
C	2.664515044365	12.153102915148	-2.910411458960
H	1.592499135365	12.246069342405	-2.695733077984
H	2.868405010875	12.619633113873	-3.883085608015
C	3.470403277970	12.810790839475	-1.825476577325
H	4.547665523732	12.784960340872	-1.963948940011
C	2.934066842716	12.948859044817	-0.456258162021
H	3.616587101142	13.030516857992	0.383379517689
H	1.948028926752	12.556251923700	-0.226076565493
O	2.936007014493	14.127943542049	-1.380738892382
O	-2.106698490079	-0.444397807082	-3.143884342822
H	-2.718028867198	0.241466960921	-3.571200805053
C	-0.660292405882	0.749229658242	-1.522949196086
H	-1.529994717960	1.395515874022	-1.374698079075
H	0.225837727505	1.405647518570	-1.504562578728
N	-0.592863698215	-0.253362039706	-0.443269090502
H	-2.917188531461	-0.668261732382	-1.681646704668
C	-0.768368607828	0.088557692893	-2.914922257017
H	-0.110199982832	-0.788590219831	-2.927102237897
C	0.794388732772	-0.638866482275	-0.070081413047
C	0.894615419539	-2.082681555157	0.456736436571
C	1.472489576765	0.346258868038	0.916025591955
H	1.366392190902	-0.605865404995	-1.010614921272
C	2.353397051680	-2.479233960937	0.767036049276
H	0.285637830507	-2.192551689716	1.363152125087
H	0.470148797442	-2.764240412136	-0.290660443132
C	2.928306507033	-0.060131551380	1.216217090099
H	1.440883429283	1.365331300617	0.507168718244
H	0.901800623200	0.364337929303	1.855461342926
C	3.034846565386	-1.505608902800	1.755352849226
H	2.368933597714	-3.500257641972	1.166973968538
H	2.936290094820	-2.497912743491	-0.168211601663
H	3.371561877857	0.640084680766	1.937458927628
H	3.528421701576	0.019356782790	0.295225653538
H	2.473988566876	-1.549254466779	2.705211839763

C	4.506201287444	-1.867696121321	2.066659059019
H	5.090090688536	-1.827626551578	1.133525135674
H	4.911205526884	-1.072317545414	2.709837205850
C	4.730989962216	-3.239652815314	2.761932172414
C	5.762915996053	-3.129845712029	3.911207745287
C	5.180916292611	-4.345232631218	1.775761004552
H	3.775380907222	-3.559695750988	3.211506080672
C	6.009082473331	-4.474519834484	4.620298136459
H	6.712992239406	-2.754861393401	3.498309081705
H	5.423526339266	-2.380627543588	4.639542632781
C	5.410654758432	-5.694253577809	2.484379534556
H	4.445751830828	-4.462214057410	0.970184776438
H	6.118725615914	-4.022007192102	1.295927855555
C	6.437267986547	-5.580699089545	3.638729159051
H	6.774284797041	-4.373759531217	5.398368232431
H	5.082167488278	-4.793587636860	5.125453759016
H	5.751421294071	-6.451163886713	1.763589165981
H	4.458610310397	-6.060316137119	2.901217373255
H	7.405741811715	-5.290476778656	3.203239018315
N	6.664132341112	-6.831341527539	4.374557518620
H	5.854189070422	-7.159519720709	4.892226035097
H	7.083354446321	-7.575010849994	3.826386060344

Structure 2

Si	-4.902130512877	2.083591312060	-2.904273462456
O	-3.361213513475	1.643402649838	-3.584819319768
H	-2.591707440416	2.241732370510	-3.707924758728
O	-5.646730177216	3.256542912756	-3.937631488185
H	-5.894712958731	3.060534694409	-4.855890321163
C	-4.689163514169	2.970478029962	-1.253822790912
H	-5.665385164401	3.248525862953	-0.844055357292
H	-4.187951013569	2.295373411329	-0.553704776445
H	-4.098036642752	3.884660296550	-1.366386219267
C	-5.845491008159	0.451461297651	-2.821672883649
H	-5.338701861666	-0.199383870432	-2.100560765443
H	-6.876388385156	0.613215792267	-2.490165295120

H	-5.865694452641	-0.048229191272	-3.795545094807
C	-1.360834095293	-0.120023061527	0.746635966330
H	-0.947941598430	-0.537231926911	1.669409714558
H	-1.580828755932	0.942227151438	0.940452914864
C	-2.699049159078	-0.834224006395	0.475091470391
H	-3.396476779577	-0.563618525871	1.274665586838
C	-2.588091295233	-2.356394804753	0.428744790432
H	-1.853855057456	-2.660598872638	-0.326558854617
H	-3.566805944874	-2.782896607638	0.181961342968
O	-2.165224302234	-2.803770208675	1.754236153384
C	-2.072908028646	-4.168477526745	2.014428887181
C	-2.300829086451	-5.170521953419	1.067205323976
C	-1.724440438289	-4.520459667658	3.328376497772
C	-2.176740359277	-6.518374079840	1.439840325291
H	-2.572354533676	-4.924020560548	0.048078289274
C	-1.608278214870	-5.860860624985	3.680168704656
H	-1.560712288279	-3.730095386568	4.052105070301
C	-1.829992509671	-6.894574565008	2.744087042797
H	-2.360013896789	-7.272119439916	0.683280465080
H	-1.356616815851	-6.116231468258	4.704808856850
C	-1.632202974516	-8.365568761401	3.177418666963
C	-0.109611645487	-8.597768093322	3.382920354003
H	0.291124769445	-7.967670792363	4.183730010743
H	0.105857431288	-9.645632762342	3.622344512272
H	0.423413818965	-8.347133480683	2.459961034421
C	-2.113013960499	-9.366241249469	2.090073714664
H	-1.989804060389	-10.390228899986	2.457700388777
H	-3.170677497400	-9.222071382676	1.846780063631
H	-1.526856396806	-9.265897524730	1.169288419793
C	-2.442650717986	-8.646854162114	4.463877805076
C	-1.898225713543	-9.273144541367	5.592246886282
C	-3.813551474609	-8.310131352804	4.505824038000
C	-2.676316609130	-9.560856705929	6.725462860034
H	-0.850763385396	-9.549424837707	5.613108440643
C	-4.600333705086	-8.590777549864	5.617484506372
H	-4.263299853709	-7.807093928834	3.655813215696

C	-4.030766652040	-9.220738011663	6.734455505968
H	-2.211158854877	-10.041138213335	7.577970515051
H	-5.651731914887	-8.329118815112	5.647492039218
O	-4.897270925098	-9.455380945378	7.803807210706
C	-4.389824882785	-10.141170318852	8.982527116356
H	-3.595796569444	-9.556505728560	9.464086051635
H	-3.981887418007	-11.121938514089	8.704830394215
C	-5.558641962504	-10.295781376584	9.914852701300
H	-6.388349493148	-10.881684765222	9.529241096497
C	-5.824807864106	-9.300743854841	10.973214516177
H	-6.833593387249	-9.175649989586	11.353112072951
H	-5.173699493661	-8.437035469729	11.070639399580
O	-3.314960633016	-0.385532990890	-0.757994100630
O	-5.182747910819	-10.608888256914	11.321905055068
C	-0.083607984342	1.039790072722	-3.939165074348
H	-0.312340451977	0.594805994948	-4.914155292938
H	0.997493077850	1.205477767628	-3.859219997686
O	-0.755010540664	2.344729482184	-3.828508211356
C	-0.264843583244	3.426650499294	-4.580307662428
C	0.670293788030	3.302305487116	-5.608436279714
C	-0.785824343802	4.685705424064	-4.251655409839
C	1.081917931355	4.448482687106	-6.307127262602
H	1.077667927789	2.337821082726	-5.885241883430
C	-0.367324113642	5.809925158049	-4.956588219666
H	-1.501741411919	4.768254473947	-3.441271909801
C	0.576750674784	5.719144045481	-6.001808528654
H	1.807838166857	4.324650725920	-7.101563815687
H	-0.765790894171	6.780964580722	-4.681684217483
C	0.962644162670	6.993903945115	-6.787184633187
C	-0.258586925068	7.395130092091	-7.660304415813
H	-1.132614036221	7.639592723938	-7.048008341599
H	-0.025891250599	8.256995525566	-8.296012331465
H	-0.529941160015	6.559363559928	-8.313394690715
C	2.161453172803	6.750982867777	-7.745330711017
H	2.422497543938	7.689459403829	-8.244802019967
H	3.047650799160	6.400961646711	-7.205997819087

H	1.911152825212	6.016699239676	-8.519720033361
C	1.367307298512	8.112818626701	-5.799998949534
C	0.839103733997	9.409006909751	-5.851771360554
C	2.351154762703	7.850277787084	-4.821465889340
C	1.265509261921	10.415948251572	-4.970807962811
H	0.079458717009	9.662196353804	-6.581535381398
C	2.787866517909	8.835675188053	-3.943063692132
H	2.770386274464	6.852188935001	-4.742248545513
C	2.244172807303	10.127336219991	-4.017004030896
H	0.824948243453	11.402966597618	-5.043689364531
H	3.541976261067	8.631120909630	-3.191933698863
O	2.740262558847	11.045490070902	-3.091346357016
C	2.256340162352	12.418019913063	-3.131565089612
H	1.178903605739	12.458571092526	-2.928019486355
H	2.442227740093	12.856339427887	-4.120766138155
C	3.014184018923	13.163033521217	-2.068684772704
H	4.092469957945	13.191852261883	-2.198308195095
C	2.458193074813	13.326328618252	-0.710142336681
H	3.127201000588	13.479926295928	0.130307045592
H	1.493375421819	12.888842650614	-0.471168613830
O	2.403317381761	14.465206953142	-1.682065981024
O	-1.874026586404	-0.430406346921	-3.050914173605
H	-2.516696257773	0.289031606706	-3.356401677871
C	-0.423512803713	0.747611778751	-1.412984055773
H	-1.279961472001	1.408397715601	-1.257166401685
H	0.476634356229	1.385955735467	-1.387122290760
N	-0.380545192222	-0.265920044631	-0.344343323060
H	-2.726809848488	-0.585436524318	-1.542976649294
C	-0.543881696539	0.110638988738	-2.815325588954
H	0.122049969709	-0.760034724231	-2.851781224049
C	0.993592208690	-0.709664773839	0.008212608602
C	1.041608756689	-2.164643591491	0.511135280325
C	1.720544375089	0.230801897495	1.003218669676
H	1.557991785741	-0.683814686945	-0.937140203786
C	2.486523982494	-2.624920729154	0.796844227308
H	0.438876392224	-2.263986104347	1.423036862119

H	0.580772059686	-2.815942690227	-0.241543990874
C	3.162518920852	-0.237881816656	1.278980284203
H	1.725163760063	1.257366583993	0.611992819533
H	1.161029732923	0.254823944506	1.949197393861
C	3.219744871242	-1.696141454850	1.791288628454
H	2.464774730845	-3.651830287804	1.180976189817
H	3.056508560517	-2.652767455009	-0.146106451314
H	3.641654811195	0.431408090640	2.006731968413
H	3.754444202150	-0.165463751320	0.352103400089
H	2.671293903328	-1.735187393059	2.748558943811
C	4.681037925174	-2.118490805658	2.074354577637
H	5.255477719731	-2.072436438451	1.135577017210
H	5.120668290812	-1.355749629247	2.734235083059
C	4.868007651130	-3.515966622851	2.728108953533
C	5.939774301104	-3.479819056449	3.845164117034
C	5.239968827969	-4.613561922851	1.700985989329
H	3.914061050173	-3.806835745154	3.200524856323
C	6.150689210871	-4.851798068852	4.512360906734
H	6.891435947375	-3.134334339166	3.410691608365
H	5.656198816859	-2.736440549581	4.602770610466
C	5.432484395989	-5.989199745742	2.368204437209
H	4.477485425700	-4.678740481664	0.915563084647
H	6.176360487759	-4.317752874463	1.201035110850
C	6.498984193453	-5.949650288285	3.490435392420
H	6.944120778365	-4.803588273162	5.266854222331
H	5.227563146775	-5.144373524107	5.040008009315
H	5.716868974597	-6.740996329904	1.618182991081
H	4.478905625464	-6.324748536369	2.806654394613
H	7.464870640741	-5.689920531023	3.030602277346
N	6.694527937055	-7.227361515738	4.187939474976
H	5.887571397991	-7.533822382062	4.723341906711
H	7.062970799842	-7.973901434483	3.608058090490

$Si(CH_3)_4$

Si	-4.886551987478	0.604267136388	-3.429194023125
C	-3.891411362225	2.068336042909	-2.680742639756

H	-4.566698882532	2.798496344165	-2.218585327743
H	-3.218673134436	1.697822723600	-1.898138990085
H	-3.307433898049	2.589831028413	-3.449326258155
C	-0.323799677584	-0.244345544700	1.095154910418
H	0.012947483182	-0.768599149501	1.993752117762
H	-0.400729933076	0.823335042296	1.351757774717
C	-1.740482919321	-0.754221109582	0.766625725701
H	-2.414937333286	-0.412721453341	1.558215836587
C	-1.845240302731	-2.275037978909	0.671927012183
H	-1.157334481834	-2.658876825662	-0.091057349165
H	-2.872614694268	-2.550283239961	0.409324190787
O	-1.498990854400	-2.819578426563	1.982766027815
C	-1.631285811544	-4.187676300642	2.209412711233
C	-2.009217268659	-5.116454025716	1.236224519631
C	-1.355811204577	-4.622202441574	3.515757574365
C	-2.109092447140	-6.474972064538	1.576046228691
H	-2.228195067918	-4.805180102647	0.222200344802
C	-1.461941877349	-5.971577594237	3.834860509004
H	-1.073254417890	-3.886202712691	4.259790243386
C	-1.840454985759	-6.932980417600	2.872507766642
H	-2.405524089674	-7.170845316976	0.800247643750
H	-1.264598764573	-6.288623277277	4.854097461816
C	-1.890172992592	-8.426077205870	3.271154518884
C	-0.428070819435	-8.909045666733	3.479647595008
H	0.062697971942	-8.371512059990	4.297571595930
H	-0.389234771236	-9.983172874349	3.695154849106
H	0.147507814177	-8.728307915898	2.565924468221
C	-2.517830731061	-9.309002091214	2.157206738530
H	-2.567315220498	-10.347521276318	2.500518515693
H	-3.535327328070	-8.988101971312	1.911681750272
H	-1.914441437115	-9.284760041223	1.242419615065
C	-2.748030741391	-8.599840897014	4.545822383838
C	-2.324763957213	-9.333254066558	5.661496744374
C	-4.044984402333	-8.042589268742	4.588537982150
C	-3.149987429866	-9.514320408403	6.783371597941
H	-1.337528118836	-9.779218174744	5.681135510794

C	-4.877314401956	-8.214613067477	5.689063551362
H	-4.397964379220	-7.453061330444	3.748508563561
C	-4.429633813581	-8.955158192693	6.793607192138
H	-2.778405968337	-10.084314551166	7.626451964917
H	-5.871313378364	-7.783615575480	5.719881715094
O	-5.332428355539	-9.067174286785	7.852602211027
C	-4.955524427452	-9.852629010831	9.018085151738
H	-4.079385454774	-9.418302950818	9.516040479312
H	-4.713833612561	-10.881434813490	8.720559275435
C	-6.141393845778	-9.830713396974	9.941399950692
H	-7.054099484816	-10.261094044233	9.538590109965
C	-6.246323007893	-8.829248601818	11.021696386713
H	-7.223158338057	-8.546094285297	11.400190478636
H	-5.461003972570	-8.088744243443	11.142156459576
O	-2.257470581920	-0.186000149751	-0.461694136642
O	-5.834256608354	-10.233816860564	11.342236525232
C	1.173424679746	1.184083629955	-3.442863912505
H	1.149848849708	0.754886138385	-4.451769319053
H	2.195797716364	1.505602055519	-3.209067319855
O	0.253172360941	2.313853807879	-3.367510198883
C	0.425955759533	3.409726140327	-4.221137516170
C	1.457087283680	3.519296713979	-5.154884201548
C	-0.516814425183	4.439182016284	-4.089117050252
C	1.535992281001	4.664845294763	-5.963325068839
H	2.198835099516	2.737941821929	-5.268968793460
C	-0.420665139827	5.567296626870	-4.897141858132
H	-1.304876826510	4.336885996308	-3.351825949228
C	0.606285399179	5.707200712281	-5.856145205570
H	2.345048737918	4.724368799873	-6.681311023588
H	-1.146922458863	6.364376353122	-4.775571608560
C	0.626194322579	6.955492791347	-6.768277609123
C	-0.559498204264	6.828353811432	-7.764539291718
H	-1.524497589559	6.817384543886	-7.247471287396
H	-0.560708039460	7.651615022073	-8.487996418730
H	-0.467425929646	5.890830699015	-8.322374048363
C	1.931432040648	7.048078165348	-7.606242105270

H	1.915804958649	7.966235499434	-8.202190891150
H	2.821329261888	7.077474387438	-6.969134053789
H	2.023590983318	6.201241459253	-8.296042986921
C	0.528844163390	8.235042901778	-5.906009647845
C	-0.387188383591	9.263435130749	-6.161046147306
C	1.430414340290	8.418465217248	-4.834731590188
C	-0.415439820407	10.435775110144	-5.388648129936
H	-1.102661329619	9.171426984922	-6.969297067992
C	1.419334457905	9.573983799924	-4.061446070287
H	2.142722732980	7.634335929503	-4.598457828993
C	0.492567095264	10.590524039252	-4.338842965224
H	-1.145406631633	11.202512468015	-5.618555208412
H	2.111125640176	9.711374271567	-3.238546681860
O	0.553942316250	11.707976073287	-3.505672588061
C	-0.348307173273	12.822761711299	-3.754943113890
H	-1.394284228971	12.513304370580	-3.635546604119
H	-0.209876709279	13.201195260269	-4.776123693598
C	-0.000696370414	13.879625298372	-2.744439211025
H	1.015227932637	14.260754568097	-2.799196507714
C	-0.725683756007	13.973698162311	-1.461097412941
H	-0.241680202714	14.425426366451	-0.601413024536
H	-1.512596913692	13.259313637929	-1.237955644816
O	-1.045138958368	14.927246831306	-2.571614691572
O	-0.741498365107	-0.126136702094	-2.729754565826
H	-1.172258478075	0.711676896714	-3.013422749313
C	0.798520687203	0.654113937899	-0.958404399812
H	0.033579532984	1.412556065837	-0.770770777549
H	1.777041268125	1.153321373360	-0.842065168745
N	0.648534003970	-0.450619407931	0.003095659714
H	-1.665236543652	-0.396597204085	-1.232122595127
C	0.660725032480	0.177966672462	-2.417143868227
H	1.196454270087	-0.767232931522	-2.543355068887
C	1.929407194561	-1.133337051764	0.331484940908
C	1.738222214164	-2.615635658257	0.702050026881
C	2.767196546031	-0.408087071796	1.415273077623
H	2.515862593058	-1.113815555242	-0.600648811091

C	3.086676520428	-3.319494438028	0.962005928402
H	1.104335845823	-2.697468520469	1.594247117622
H	1.202194024693	-3.118873168612	-0.112067959056
C	4.111981300816	-1.118907940139	1.663266714040
H	2.940601082795	0.635589030316	1.119246380206
H	2.195406930124	-0.380192731363	2.353568203000
C	3.928976970190	-2.606593950915	2.044282887817
H	2.896307522512	-4.359663411496	1.252320316142
H	3.669508900626	-3.354437077882	0.027182772410
H	4.670528850834	-0.598188245522	2.453057583193
H	4.730837811221	-1.060230170757	0.753182056077
H	3.356310427658	-2.641245808996	2.987361170757
C	5.298477949579	-3.276798229265	2.306210761049
H	5.896160047658	-3.243983291805	1.381513530587
H	5.835784064009	-2.651449250034	3.034663989144
C	5.246310149951	-4.736153111560	2.838601486569
C	6.271931367875	-4.960941732874	3.976780217526
C	5.479813454409	-5.788398448973	1.726783153658
H	4.243511916200	-4.913271137228	3.263724870821
C	6.246515961595	-6.399958035600	4.524970733464
H	7.279700181707	-4.733068967042	3.594410078798
H	6.079987403095	-4.248314750517	4.790651219150
C	5.436614706837	-7.228263400103	2.274261998445
H	4.742857806774	-5.667802096596	0.923508256985
H	6.466588605763	-5.601150628810	1.273561188909
C	6.457539268607	-7.449154310763	3.417920026150
H	7.011305299060	-6.539317982337	5.297504912003
H	5.271732694455	-6.588243268793	5.005024902297
H	5.628140285040	-7.949637226255	1.467073799179
H	4.428503943328	-7.447063771661	2.661484689926
H	7.466991962058	-7.305244015752	3.003076985314
N	6.431039883725	-8.795549515315	4.004056940035
H	5.568724096278	-9.017115425328	4.492919006731
H	6.702078211103	-9.539129698597	3.369280045118
C	-6.343449832867	1.286829371848	-4.482434673370
H	-7.027822182513	1.879454860301	-3.864005191077

H	-5.975769051058	1.928108117295	-5.292568204438
H	-6.916884702409	0.467866356154	-4.932540937115
C	-3.743747631200	-0.437080698105	-4.571659258610
H	-4.278973117711	-1.314395585603	-4.954478429243
H	-3.414747944877	0.156984679790	-5.433461362385
H	-2.852650788724	-0.789119118463	-4.039748413660
C	-5.559275296018	-0.485450296213	-2.003090446182
H	-4.731828226887	-0.764459329809	-1.341424771355
H	-6.299782539170	0.063204069730	-1.409119973956
H	-6.036359003163	-1.395520737110	-2.384978410342

$(CH_3)_2SiF_2$

Si	-4.055956011503	1.903650237875	-4.011095261265
C	-3.855625991350	2.473419868703	-2.241066313150
H	-4.749948469754	3.024298982099	-1.929280742897
H	-3.693046462867	1.645741472022	-1.539583142270
H	-2.990752592039	3.139654645804	-2.168515925922
C	-0.622862210521	0.032528150490	1.094205292129
H	-0.264927940720	-0.489155472790	1.985981014179
H	-0.668442599999	1.105947278452	1.339921910405
C	-2.062594157559	-0.449717201032	0.824490981402
H	-2.692288044142	-0.115713913719	1.655955736202
C	-2.184764242445	-1.967358138347	0.703478904105
H	-1.524818432634	-2.336103038559	-0.090468364518
H	-3.222892853761	-2.229423626427	0.470798586204
O	-1.797971901327	-2.543534825079	1.990662894806
C	-1.887394513892	-3.920096078866	2.173553119258
C	-2.283739942754	-4.824642650316	1.184345361565
C	-1.547878661156	-4.393463727832	3.451429753353
C	-2.335228452788	-6.196248911683	1.478739541483
H	-2.552927137407	-4.483769294148	0.192218820645
C	-1.606700370075	-5.755517791633	3.725767287966
H	-1.252153586047	-3.676794611500	4.209205990204
C	-2.000405265598	-6.692479685529	2.745483660204
H	-2.646546458518	-6.871973768765	0.690941452133
H	-1.358246757934	-6.102674729375	4.723891959453

C	-1.991835482309	-8.199803239815	3.089639938786
C	-0.509208582284	-8.648312942914	3.213265516593
H	0.003970107301	-8.127443228608	4.028291769208
H	-0.431216877627	-9.728208579952	3.385233458951
H	0.018446730462	-8.416994225054	2.282240293092
C	-2.646563247765	-9.059280115613	1.972698717865
H	-2.653086552306	-10.110553236567	2.278791425346
H	-3.682184057125	-8.757869151643	1.784997881174
H	-2.086563377631	-8.985141079329	1.033382821078
C	-2.784627266970	-8.445516614438	4.394178216955
C	-2.291211054844	-9.209376114723	5.459485276617
C	-4.092405164932	-7.926963552875	4.517726133023
C	-3.058786870244	-9.457354378568	6.609132164756
H	-1.292618710088	-9.627550498919	5.416762334294
C	-4.868431321371	-8.165417577342	5.646685869614
H	-4.498834489572	-7.315041290999	3.718891054394
C	-4.351072565172	-8.935789286631	6.699115430182
H	-2.633641110050	-10.048880889945	7.411110563059
H	-5.871023035049	-7.764195091429	5.739380467470
O	-5.201242895173	-9.115503064585	7.792199973714
C	-4.750529418519	-9.936536284623	8.905541717618
H	-3.865808301117	-9.496925677197	9.383220337704
H	-4.493093429580	-10.944354187123	8.554268664328
C	-5.894183269010	-9.988691737298	9.879439281134
H	-6.810833487638	-10.430496591223	9.498830620831
C	-5.980460270179	-9.035203910323	11.003827245197
H	-6.947399025945	-8.797735756512	11.435314826353
H	-5.212582698331	-8.276274136821	11.120964573950
O	-2.624922033755	0.145051350802	-0.370013312969
O	-5.513976172905	-10.437968386583	11.247955884302
C	0.437106588616	1.260798131788	-3.584039384721
H	0.135541981450	0.859751623581	-4.560194970169
H	1.512402907383	1.481401264998	-3.611087188059
O	-0.302035813211	2.488252702777	-3.306222253594
C	-0.134127591128	3.579798801393	-4.160843824264
C	0.587761046425	3.544430424942	-5.356632675254

C	-0.751017294462	4.774818123477	-3.756449680915
C	0.680005147032	4.702089900085	-6.145680326256
H	1.076811401650	2.637970597934	-5.691052684823
C	-0.647848213184	5.912821960419	-4.551011675312
H	-1.280383154732	4.797145715389	-2.810542347739
C	0.068119170366	5.904395170531	-5.767877043604
H	1.245340074868	4.642697597653	-7.067885986603
H	-1.111443032454	6.834220264572	-4.213326659921
C	0.102878188434	7.182783088168	-6.636774598240
C	-1.314698325815	7.379453190159	-7.242250237363
H	-2.069615689690	7.536211933125	-6.464764360828
H	-1.339255680768	8.233727732885	-7.928276967015
H	-1.595849273275	6.485884167484	-7.809138558013
C	1.101972101974	7.058802235362	-7.820279506819
H	1.120559762472	7.999620450170	-8.379618959138
H	2.119935307028	6.856253597115	-7.471906040882
H	0.804646165715	6.262226133423	-8.512089312639
C	0.541029969968	8.389161559129	-5.774644064432
C	-0.156289072677	9.603549915242	-5.744411198401
C	1.726413189902	8.301009906367	-5.012046037755
C	0.297661004162	10.697331641064	-4.989802463688
H	-1.072661654232	9.723354157014	-6.309819874559
C	2.192764632339	9.374638783404	-4.261658777904
H	2.283398755090	7.369485603104	-4.999606214749
C	1.476597904501	10.581496046261	-4.249822443846
H	-0.276955971856	11.615733977326	-4.992053999568
H	3.102065073710	9.303799010233	-3.676130603591
O	2.019178958259	11.598264262324	-3.464045951882
C	1.358548163522	12.895366885086	-3.435307326805
H	0.348205600687	12.809997101120	-3.015945383275
H	1.282243329176	13.304095952659	-4.451311846205
C	2.207672778978	13.781658524531	-2.567875392089
H	3.223759158361	13.941344252788	-2.917620308024
C	1.920039896245	13.938885408937	-1.127778291109
H	2.717773255971	14.217377542467	-0.446966766694
H	1.091202198421	13.394175646101	-0.685464887269

O	1.517324760655	15.012601107196	-2.091929728298
O	-1.210144396861	-0.285305034141	-2.580358564611
H	-1.700202202014	0.039620053456	-3.370510828675
C	0.356410540709	0.817977514222	-1.068571981723
H	-0.425422889782	1.558053880956	-0.887011729876
H	1.320873046411	1.356551767857	-1.053947565970
N	0.305202053891	-0.216450860888	-0.024811782216
H	-2.092231474206	-0.119659906044	-1.175517267875
C	0.145763460871	0.247017095571	-2.482341965441
H	0.833185708704	-0.596911156558	-2.634818273489
C	1.615533748725	-0.844996845192	0.279831647241
C	1.480146445944	-2.307195641634	0.745747020104
C	2.482409781479	-0.035392147927	1.278183247890
H	2.157575138000	-0.867785965058	-0.678586286595
C	2.856346887959	-2.961603528123	0.988537510085
H	0.886764246698	-2.350876188441	1.668127674890
H	0.921603704520	-2.870224243206	-0.011774964227
C	3.854097174810	-0.698724034606	1.508891962596
H	2.615697474791	0.991208672629	0.910113703389
H	1.950691619211	0.038602186319	2.237504535864
C	3.724126362400	-2.163342087536	1.988140792119
H	2.706930909592	-3.986685275066	1.348770887996
H	3.400463892526	-3.039354067415	0.033117090529
H	4.433789364983	-0.117928304417	2.239581487615
H	4.431152744315	-0.683488330192	0.569971141310
H	3.191451283635	-2.151175493927	2.955026776908
C	5.118757385481	-2.786192641696	2.233048454238
H	5.673209166739	-2.808655464377	1.281540987347
H	5.674600408644	-2.100267610853	2.889560891108
C	5.121601829583	-4.204170922038	2.869931672985
C	6.188324345178	-4.321636994374	3.986222948449
C	5.345295401944	-5.328768598134	1.829526858269
H	4.137756604625	-4.373408292105	3.340165959918
C	6.216848070183	-5.716862228094	4.637440037351
H	7.177325151357	-4.099287282474	3.554659125713
H	6.004410601344	-3.555980498505	4.752356432275

C	5.356963522568	-6.725448688714	2.480694414850
H	4.578715221398	-5.283582578037	1.046353674235
H	6.311248783135	-5.152433653828	1.329433169970
C	6.419226190771	-6.838466527184	3.602330981635
H	7.009101081122	-5.782007168829	5.391933001495
H	5.262856262874	-5.892024058318	5.162211242170
H	5.541078342029	-7.499222172944	1.721756899550
H	4.367702619220	-6.938860378947	2.916539762744
H	7.410976915053	-6.701599756631	3.144557437707
N	6.445929089268	-8.139116471744	4.284366435439
H	5.605625547505	-8.343409376451	4.817261560177
H	6.712467427002	-8.920416384880	3.694495566727
C	-4.165816098728	3.217252877326	-5.349735766366
H	-5.048821211013	3.845671939210	-5.193763762276
H	-3.276540138463	3.855064487774	-5.326788853915
H	-4.241783655629	2.758860419864	-6.339716835114
F	-2.750085367025	0.869469858993	-4.447353257080
F	-5.392256743249	0.869512122465	-4.150276366182
